# Cybervictimization and Academic Achievement: The Unique Buffering Role of Academic and Social Teacher–Student Relationships

**DOI:** 10.3390/children13101347

**Published:** 2026-10-04

**Authors:** Ahmed Al Saidi, Laura Badenes-Ribera, Antonio Ferrer, Watfa Al Mamari, Michele Settanni, Claudio Longobardi, Sofia Mastrokoukou

**Affiliations:** 1 Department of Behavioral Sciences Methodology, Faculty of Psychology, University of Valencia, 46010 Valencia, Spain; laura.badenes@uv.es; 2 Department of Child Health, College of Medicine and Health Sciences, Sultan Qaboos University, P.O. Box 50, Al-Khoudh P.C. 123, Muscat, Oman; 3 Department of Child Health, Sultan Qaboos University Hospital, University Medical City, P.O. Box 112, Al-Khoudh P.C. 123, Muscat, Oman; 4 Department of Developmental and Educational Psychology, Faculty of Psychology, University of Valencia, 46010 Valencia, Spain; antonio.ferrer@uv.es; 5 Department of Psychology, University of Turin, 10123 Turin, Italy; michele.settanni@unito.it (M.S.); claudio.longobardi@unito.it (C.L.); 6 Department of Human and Social Sciences, Universitas Mercatorum, 00186 Rome, Italy; sofia.mastrokoukou@unimercatorum.it

**Keywords:** cybervictimization, academic achievement, teacher–student relationships, instructional support, adolescence, moderation, cross-cultural research, Oman

## Abstract

**Highlights:**

**What are the main findings?**

- Academic teacher–student relationships buffered the negative association between cybervictimization and academic achievement.
- Social teacher–student relationships did not show a unique buffering effect when academic support was considered simultaneously.

**What are the implications of the main findings?**

- Instructional support, structure, and feedback from teachers may help preserve academic functioning among cybervictimized adolescents.
- School-based interventions should distinguish academic from socio-emotional teacher support and consider their potentially different protective roles.

**Abstract:**

**Background/Objectives:** Cybervictimization is a pervasive developmental stressor with potential consequences for adolescents’ academic functioning. Drawing on stress-buffering and domain-specific motivation frameworks, the present study examined whether distinct dimensions of teacher–student relationships (TSR)—academic (instructional support) and social (socio-emotional support)—uniquely moderate the association between cybervictimization and grade point average (GPA). **Methods:** Participants were 1458 Omani adolescents (M_age = 14.04 years; 50% female) recruited from secondary schools. Students completed validated self-report measures of cybervictimization and TSR and reported their GPA. Moderation analyses were conducted using PROCESS v4.2 (Model 3), simultaneously testing academic and social TSR as moderators while adjusting for sex, age, father education, mother education, and household income. **Results:** Cybervictimization showed a small negative zero-order association with GPA; however, its conditional effect at the mean levels of both TSR dimensions did not reach conventional statistical significance. Academic TSR was positively associated with GPA and significantly buffered the negative link between cybervictimization and academic performance, such that the association was strongest at low levels of instructional support and attenuated at higher levels. In contrast, social TSR did not uniquely moderate the association once academic support was included in the model, and the three-way interaction was not significant. The local effect size for the Cybervictimization × Academic TSR interaction was small (f^2^ = 0.033). **Conclusions:** Findings support a domain-specific buffering hypothesis, although the incremental protective effect was modest. Results extend resilience models beyond Western contexts and highlight the centrality of academic-focused teacher support in promoting achievement in rapidly digitalizing educational systems.

## 1. Introduction

Adolescence is a critical developmental period characterized by heightened sensitivity to peer evaluation and increasing reliance on digitally mediated social interactions. In this context, cybervictimization-peer aggression enacted through digital communication technologies-has become a prevalent and developmentally salient stressor [1,2,3]. Compared to traditional bullying, cybervictimization can be persistent, potentially anonymous, and rapidly amplified to large audiences, extending peer harassment beyond school grounds and into settings that are typically experienced as private or safe [4,5]. Meta-analytic and review evidence indicates that a substantial proportion of adolescents experience cybervictimization and that such exposure is reliably associated with elevated stress and internalizing symptoms, as well as sleep disruption [4,6]. Importantly, these mental health correlates are not only clinically meaningful; they may also undermine core functional domains central to adolescent development, including school adjustment and academic performance.

Academic achievement, often indexed by grade point average (GPA), represents a key functional outcome with long-term implications for educational and occupational trajectories. Although the psychological correlates of cybervictimization are increasingly well documented, clarifying its academic implications remains essential because school performance is a primary developmental task during adolescence and a major predictor of later life opportunities. Cybervictimization may compromise academic performance through multiple interrelated pathways. Exposure to digital peer aggression can increase rumination, threat vigilance, and emotional arousal, which consume cognitive resources required for learning and sustained attention [7]. Cybervictimization is also associated with sleep disruption and stress-related complaints [4], which can compromise executive functioning, concentration, and classroom readiness—processes with direct relevance for achievement. When cybervictimization is embedded within school-based peer networks, adolescents may perceive school as socially unsafe, prompting avoidance, reduced participation, and disengagement from academic tasks [3]. Finally, chronic peer aggression can erode academic self-efficacy and task persistence, weakening motivational resources necessary for maintaining performance under adversity [8]. Together, these mechanisms position GPA as a meaningful downstream indicator of functional impairment associated with bullying-related stress, even when mental health symptoms are not directly measured.

At the same time, not all cybervictimized adolescents show comparable academic impairment, suggesting meaningful variability in vulnerability and protection processes. Identifying school-based protective factors capable of buffering academic functioning under cybervictimization is therefore both a developmental and applied priority. Bioecological perspectives emphasize that development is shaped by repeated, reciprocal interactions within proximal contexts, with teacher–student relationships (TSR) representing a central microsystem for adolescents [9]. Supportive TSR have been consistently linked to engagement, school belonging, and academic outcomes [10,11,12]. From a stress-buffering perspective, supportive adult relationships may reduce the functional toll of stressors by promoting adaptive coping, stabilizing students’ sense of safety, and maintaining engagement with school goals [13]. Accordingly, TSR are theoretically plausible moderators of the association between cybervictimization and academic performance.

A critical gap in the literature is that TSR are often operationalized using broad, global indices of warmth and support. However, relationship quality and classroom interaction research suggests separable functional dimensions—particularly academic/instructional and socio-emotional components—that may carry different implications depending on the outcome [14,15,16]. Academic TSR reflects instructional support processes (e.g., scaffolding, monitoring, feedback, classroom structure) that directly shape learning opportunities and performance, whereas social TSR captures affective qualities such as care, trust, and emotional support. Because these dimensions are typically correlated, examining them separately can obscure which aspects of TSR contribute uniquely to resilience [17]. A simultaneous moderation framework provides a stricter test by estimating the unique buffering contributions of academic and social TSR within the same model.

Drawing on domain-specific models of motivation and achievement, protective processes are expected to be strongest when the support provided matches the functional domain of the outcome [8,12]. GPA reflects performance in instructional tasks that depend on sustained attention, feedback utilization, and structured learning conditions. Academic TSR—through clear expectations, scaffolding, and actionable feedback—may therefore be particularly well positioned to buffer cybervictimization-related decrements in performance by helping students remain oriented toward learning goals despite stress-related cognitive and motivational disruption. By contrast, social TSR may be more proximal to mental health outcomes (e.g., internalizing symptoms, belonging) and may not uniquely protect GPA once instructional support is accounted for. Testing these dimensions simultaneously can therefore clarify whether protective effects for academic performance are uniquely attributable to academic TSR rather than to broader socio-emotional support.

Most evidence on cybervictimization and TSR derives from WEIRD educational contexts [18,19]. Examining these processes in Oman—a rapidly digitalizing non-Western context with distinct educational norms and teacher authority dynamics—offers a valuable test of the cross-cultural generalizability of school-based resilience models. Such evidence is important for establishing whether domain-specific TSR buffering represents a robust developmental process across sociocultural settings. To address these gaps, the present study examined the association between cybervictimization and academic performance (GPA) in a large sample of Omani adolescents and tested academic and social TSR as simultaneous moderators while adjusting for key sociodemographic covariates (sex, age, father education, mother education, and household income). The hypothesized simultaneous moderation framework is illustrated in Figure 1. We hypothesized that higher cybervictimization would be associated with lower GPA (H1) and that academic TSR would buffer this association, such that the negative association would be weaker at higher levels of academic TSR (H2). Given conceptual overlap between TSR dimensions, we expected social TSR to show reduced or non-significant unique moderation once academic TSR was included (H3). Finally, we explored whether a three-way interaction (Cybervictimization × Academic TSR × Social TSR) emerged, without strong directional expectations.

## 2. Methods

### 2.1. Participants

Participants were 1458 adolescents recruited from secondary schools in Oman. The analytic sample reflected early–mid adolescence (*M*_age_ = 14.04 years, *SD* = 0.20, range = 14–15). Approximately half of the sample was female (Sex01: *M* = 0.50, *SD* = 0.50; coded 0 = male, 1 = female). Descriptive statistics for focal variables are reported in Table 1.

### 2.2. Procedure

Data were collected in secondary schools in Oman using a school-based survey design. After obtaining institutional approvals and school permissions, eligible students were invited to participate through their schools. Information sheets were provided to students and their caregivers/guardians, and written informed consent was obtained prior to participation.

Questionnaires were administered during the school day in a classroom setting by trained research personnel (with support from school staff as needed). Students were informed that participation was voluntary, that responses would be kept confidential and used only for research purposes, and that they could skip any question or discontinue at any time without consequences. Standardized classroom administration and anonymous responding were used to reduce evaluation apprehension and social desirability, which are particularly relevant when assessing experiences of peer aggression. Surveys were completed anonymously using unique study IDs, and no identifying information was included in the analytic dataset.

The research plan was approved by the authors’ affiliated university (n. 0592463). The study strictly adhered to the ethical principles outlined in the Declaration of Helsinki, with all participants treated in accordance with ethical guidelines for research involving human subjects.

### 2.3. Measures

#### 2.3.1. Academic Performance (GPA)

Academic performance was indexed using self-reported GPA, which was recoded into a 5-point scale consistent with the Omani educational assessment system (1 = Not accepted/Fail to 5 = Excellent). Higher scores reflected superior academic achievement across core subjects. Self-reported grades have shown substantial correspondence with official grades in adolescent samples, although modest over-reporting can occur and accuracy varies across students and contexts [20,21]. Accordingly, the present GPA measure is treated as a pragmatic group-level indicator of academic performance rather than as a substitute for verified school records.

#### 2.3.2. Cybervictimization

Cybervictimization was assessed using the victimization subscale of the Revised Cyber Bullying Inventory–II (RCBI-II) [22]. This scale consists of 10 items describing various forms of digital aggression (e.g., “Someone sent me threatening or insulting messages via the internet,” “Someone posted embarrassing photos of me online”). Adolescents rated the frequency of these experiences over the previous six months on a 4-point Likert scale (1 = Never, 4 = More than three times). Total scores were calculated by summing responses, with higher scores indicating more frequent cybervictimization. The RCBI-II has demonstrated strong psychometric properties in diverse adolescent populations. Internal consistency was good (α = 0.88).

#### 2.3.3. Teacher–Student Relations (TSR)

Teacher–student relations were assessed using the 25-item Student–Teacher Relationship Measure (S-TRM), developed and validated with Omani students in Grades 7–11 [23]. The measure comprises two correlated subscales: Academic Relations (15 items) and Social Relations (10 items). The Academic Relations subscale captures teachers’ instructional characteristics, including learning support, classroom management, and motivation (e.g., “My teacher expects me to participate effectively in the classroom”; “My teacher gives some hints to provide the right answer”). The Social Relations subscale assesses teachers’ personal and relational characteristics, including caring, trust, and a safe learning environment (e.g., “My teacher listens to what I say”; “My teacher cares about my academic performance”). Responses were provided on a 5-point scale (1 = Definitely does not apply, 5 = Definitely applies). Item responses were summed within each subscale, yielding possible ranges of 15–75 for Academic Relations and 10–50 for Social Relations; higher scores indicate more positive teacher–student relations in the respective domain. Reliability was excellent (Academic α = 0.91; Social α = 0.89).

#### 2.3.4. Covariates

To isolate the effects of the focal predictors, we adjusted for several sociodemographic variables: sex (0 = male, 1 = female), age, father education, mother education, and household income. Parental education and income were included as socioeconomic indicators because they may covary with both academic performance and adolescents’ school experiences.

### 2.4. Analytic Strategy

Primary hypotheses were tested using ordinary least squares regression with moderation analyses conducted in PROCESS v4.2 (Model 3) [24]. GPA was specified as the outcome (Y), cybervictimization as the focal predictor (X), and academic and social teacher–student relations as simultaneous income moderators (W and Z). Sex, age, father education, mother education, and household were included as covariates.

Continuous variables were mean-centered prior to creating product terms. Hypothesis tests and significance statements were based on OLS coefficient estimates, standard errors, *t* tests, *p* values, and model-based 95% confidence intervals. Percentile bootstrap estimates based on 5000 resamples were examined as a supplementary robustness check and were not used to override model-based significance tests when the two inferential approaches differed. Missing data were handled using listwise deletion for cases with missing values on any variable included in the model. To improve effect-size interpretability, local Cohen’s f^2^ was calculated for each 1-df interaction test (f^2^ = t^2^/dferror); partial R^2^ is also reported for the focal Cybervictimization × Academic Relations interaction.

## 3. Results

### 3.1. Descriptive Statistics and Pearson’s Correlations

Descriptive statistics and zero-order correlations are reported in Table 1. On average, adolescents reported relatively high academic performance (*GPA*, 1–5; *M* = 4.47, *SD* = 0.93), moderate levels of cybervictimization (*M* = 12.15, *SD* = 3.92), and moderate-to-high teacher–student relationship quality in both the academic (*M* = 50.92, *SD* = 12.93) and social (*M* = 35.24, *SD* = 8.87) domains.

Coefficients are interpreted conditional on other covariates and TSR; multicollinearity diagnostics (VIF) were within acceptable ranges. At the bivariate level, cybervictimization was negatively associated with GPA, *r* = −0.13, whereas academic teacher–student relations were positively associated with GPA, *r* = 0.06. Social teacher–student relations were not meaningfully associated with GPA, *r* = −0.01. Academic and social relations were strongly correlated, *r* = 0.71, indicating substantial shared variance between the two teacher–student relationship dimensions. Multicollinearity diagnostics were nevertheless within conventional limits (all VIFs ≤ 2.10; tolerances ≥ 0.476). Because the moderators share considerable variance, coefficients from the simultaneous model represent unique conditional contributions after shared variance is partitioned; possible suppression or instability of the unique coefficients is considered in the Discussion.

### 3.2. Moderated Regression Model

To examine whether academic and social teacher–student relations simultaneously moderated the association between cybervictimization and GPA, we estimated a moderated regression using PROCESS v4.2 (Model 3) [24]. Continuous predictors were mean-centered prior to the computation of interaction terms, and cases with missing data on variables included in the model were handled using listwise deletion.

The overall model was statistically significant, R^2^ = 0.093, adjusted R^2^ = 0.086, F(12, 1445) = 12.39, *p* < 0.001 (see Table 2). Given the substantial correlation between the academic and social TSR dimensions, the primary inferential focus was on their unique moderating contributions. Specifically, each interaction was evaluated while simultaneously accounting for the other TSR dimension, their shared variance, and all included covariates.

### 3.3. Main Effects

Controlling for covariates and interaction terms, academic teacher–student relations were significantly and positively associated with GPA at the centered levels of cybervictimization and social teacher–student relations, *b* = 0.0074, *SE* = 0.0026, *t* = 2.80, *p* = 0.005, 95% CI [0.0022, 0.0125]. In contrast, social teacher–student relations were not significantly associated with GPA under the corresponding conditional interpretation, *b* = −0.0034, *SE* = 0.0039, *t* = −0.88, *p* = 0.380, 95% CI [−0.0110, 0.0042].

The conditional effect of cybervictimization on GPA at the centered levels of both teacher–student relationship dimensions was not statistically significant, *b* = −0.0147, *SE* = 0.0077, *t* = −1.91, *p* = 0.057, 95% CI [−0.0299, 0.0004]. Thus, this coefficient does not provide evidence of a statistically significant conditional effect of cybervictimization on GPA. Regarding the covariates, sex, age, and father’s education were not significantly associated with GPA, whereas mother’s education and household income showed significant associations with GPA (see Table 2).

### 3.4. Two-Way and Three-Way Interactions

As hypothesized, the Cybervictimization × Academic relations interaction was significant and positive, *b* = 0.0046, *SE* = 0.0007, *t* = 6.85, *p* < 0.001, 95% CI [0.0033, 0.0059]. The interaction had partial R^2^ = 0.031 and local f^2^ = 0.033, indicating a small incremental effect. This pattern indicates that higher academic teacher–student relations attenuated (buffered) the negative association between cybervictimization and GPA, although the magnitude of this effect should be interpreted as modest rather than as evidence of a large protective effect.

In contrast, the Cybervictimization × Social Relations interaction was not statistically significant, *b* = −0.0008, *SE* = 0.0011, *t* = −0.79, *p* = 0.433, 95% CI [−0.0030, 0.0013], local *f*^2^ < 0.001, suggesting that social teacher–student relations did not account for unique moderation once academic relations and their shared variance were modeled simultaneously. Because academic and social relations were strongly correlated, this nonsignificant unique interaction should not be interpreted as evidence that socio-emotional teacher–student relationships are unimportant more generally.

The Cybervictimization × Academic Relations × Social Relations three-way interaction was also not statistically significant, Δ*R*^2^ = 0.0016, *F*(1, 1445) = 2.61, *p* = 0.106, local *f*^2^ = 0.002, indicating no evidence that the moderating role of academic teacher–student relations depended on the level of social teacher–student relations.

To facilitate interpretation of the significant Cybervictimization × Academic TSR interaction, predicted GPA was plotted at low (−1 SD), mean, and high (+1 SD) levels of academic TSR, holding social TSR at its mean and covariates constant (Figure 2). The negative association between cybervictimization and GPA was strongest at low academic TSR and progressively weaker as academic TSR increased, consistent with an academic-specific buffering effect.

## 4. Discussion

The present study examined whether cybervictimization is associated with academic performance (GPA) in a large sample of Omani adolescents and whether distinct dimensions of teacher–student relationships (TSR) uniquely buffer this association. Cybervictimization is widely documented as a mental health-relevant stressor linked to distress, internalizing symptoms, and sleep disruption [4,6,25]. From a functional perspective, these correlates can plausibly translate into academic impairment via reduced cognitive efficiency, compromised self-regulation, and school disengagement. In the present study, cybervictimization showed a small negative zero-order association with GPA, but its conditional coefficient at the mean levels of the two TSR dimensions did not reach conventional significance in the full moderated model. Critically, the cybervictimization–GPA association varied as a function of Academic Relations: adolescents reporting stronger academic relationships with teachers showed a weaker negative association between cybervictimization and academic performance. This interaction remained evident when Social Relations and sociodemographic covariates were included simultaneously.

Consistent with stress-buffering accounts [13] and domain-specific achievement frameworks [8,12], academic TSR uniquely moderated the association between cybervictimization and GPA, whereas social TSR did not provide incremental buffering once academic TSR was included. This pattern supports a domain-specific buffering interpretation: when cybervictimization-related distress and vigilance threaten attention and persistence, teachers’ instructional scaffolding, feedback, and classroom structure may help adolescents remain oriented toward academic tasks, thereby protecting performance outcomes. In contrast, socio-emotional closeness may be especially consequential for mental health outcomes (e.g., internalizing symptoms, belonging), but appears less uniquely predictive of GPA when instructional support is considered simultaneously. This interpretation remains correlational, however, and should not be read as evidence that stronger academic TSR causally prevents academic harm.

### 4.1. The Specificity of the Buffering Effect: Academic vs. Social TSR

The most significant contribution of this study lies in the simultaneous moderation test. Academic and social TSR were strongly correlated (r = 0.71), so their coefficients should be interpreted as unique conditional contributions after substantial shared variance was partitioned. The Academic interaction remained significant (local f^2^ = 0.033), whereas the Social interaction did not (local f^2^ < 0.001). Although this pattern supports functional differentiation between the two dimensions, the focal interaction was small by conventional benchmarks, and the null unique Social Relations interaction should not be interpreted as evidence that socio-emotional teacher relationships are unimportant more generally. The non-significant three-way interaction indicated no evidence that the Academic Relations moderation varied across levels of Social Relations.

### 4.2. Cross-Cultural Generalizability in the Omani Context

By conducting this research in Oman, we addressed the “WEIRD” bias in adolescent psychology [18,19]. Omani schools often emphasize teacher authority and structured learning environments. Our findings suggest that the academic buffering pattern can also be observed in a non-Western, rapidly digitalizing society, where the teacher remains a proximal adult in the adolescent’s bioecological system [9]. This highlights the importance of school ecology as a site for further cross-cultural research and intervention development.

## 5. Limitations and Future Directions

Despite the strengths of this study—including its large sample size and the use of a simultaneous moderation framework—several limitations must be noted. First, the cross-sectional nature of the data precludes definitive causal conclusions. While we modeled cybervictimization as the predictor and GPA as the outcome, it is possible that academic struggles could increase vulnerability to peer victimization. Longitudinal designs are needed to establish the temporal precedence of these associations. Second, cybervictimization, TSR, and GPA were all reported by the same adolescents. This common-source design may inflate or otherwise distort associations through shared method variance, response style, recall, or social-desirability processes. Future research would benefit from incorporating teacher ratings or observational indicators of relationship quality and verified school records. Third, GPA was measured as a self-reported five-category indicator. Self-reported grades often correspond substantially with official records but can show modest over-reporting and individual-level error [20,21]; replication using verified continuous grade records and sensitivity analyses with ordinal outcome models would strengthen inference. Fourth, academic and social TSR were highly correlated (r = 0.71). Although VIFs were acceptable, the strong overlap means that unique coefficients may be sensitive to how shared TSR variance is partitioned; therefore, the null unique Social Relations interaction should not be generalized to socio-emotional teacher support across other outcomes or measurement models. Finally, the focal interaction had a small local effect size (f^2^ = 0.033), so practical implications should be framed as modest and hypothesis-generating rather than as evidence of a large intervention effect. Future studies should also examine whether social TSR might play a more dominant role for other outcomes, such as depression, anxiety, belonging, or school-based social withdrawal.

## 6. Practical Implications and Conclusions

Our findings have potential implications for school-based interventions and teacher professional development. The results suggest that “supportive teaching” should not be viewed only through an emotional lens. For students experiencing cybervictimization, maintaining high-quality instructional practices, providing clear structure, scaffolding difficult tasks, and offering constructive feedback, may represent one school-based resource associated with better preservation of academic functioning. However, the observed interaction was small, the data were cross-sectional, and all focal measures were self-reported; the findings therefore do not establish that increasing academic TSR alone will produce sizeable gains in GPA.

In conclusion, cybervictimization showed a small negative zero-order association with academic achievement in Omani adolescents, and this association varied as a function of academic TSR. When academic and social TSR were modeled simultaneously, only the academic dimension showed unique moderation of the cybervictimization–GPA association. The finding was modest and correlational, but it identifies academic-oriented teacher support as a potentially relevant resource for academic resilience in the context of digital peer aggression and provides a basis for longitudinal and intervention research in non-Western educational settings.

## Figures and Tables

**Figure 1:**
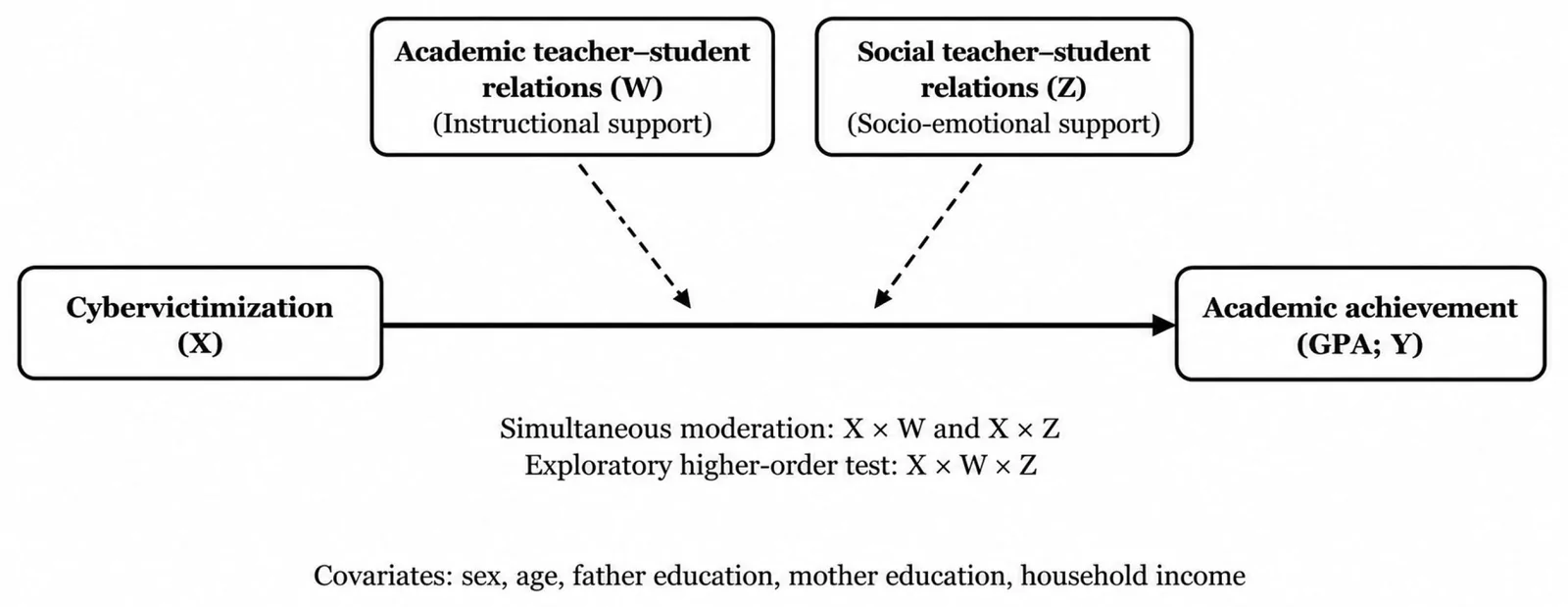
Conceptual Model of the Hypothesized Simultaneous Moderation Framework. Note. Covariates are shown for completeness. The Academic TSR × Social TSR contribution to the cybervictimization slope was examined exploratorily through the three-way interaction.

**Figure 2:**
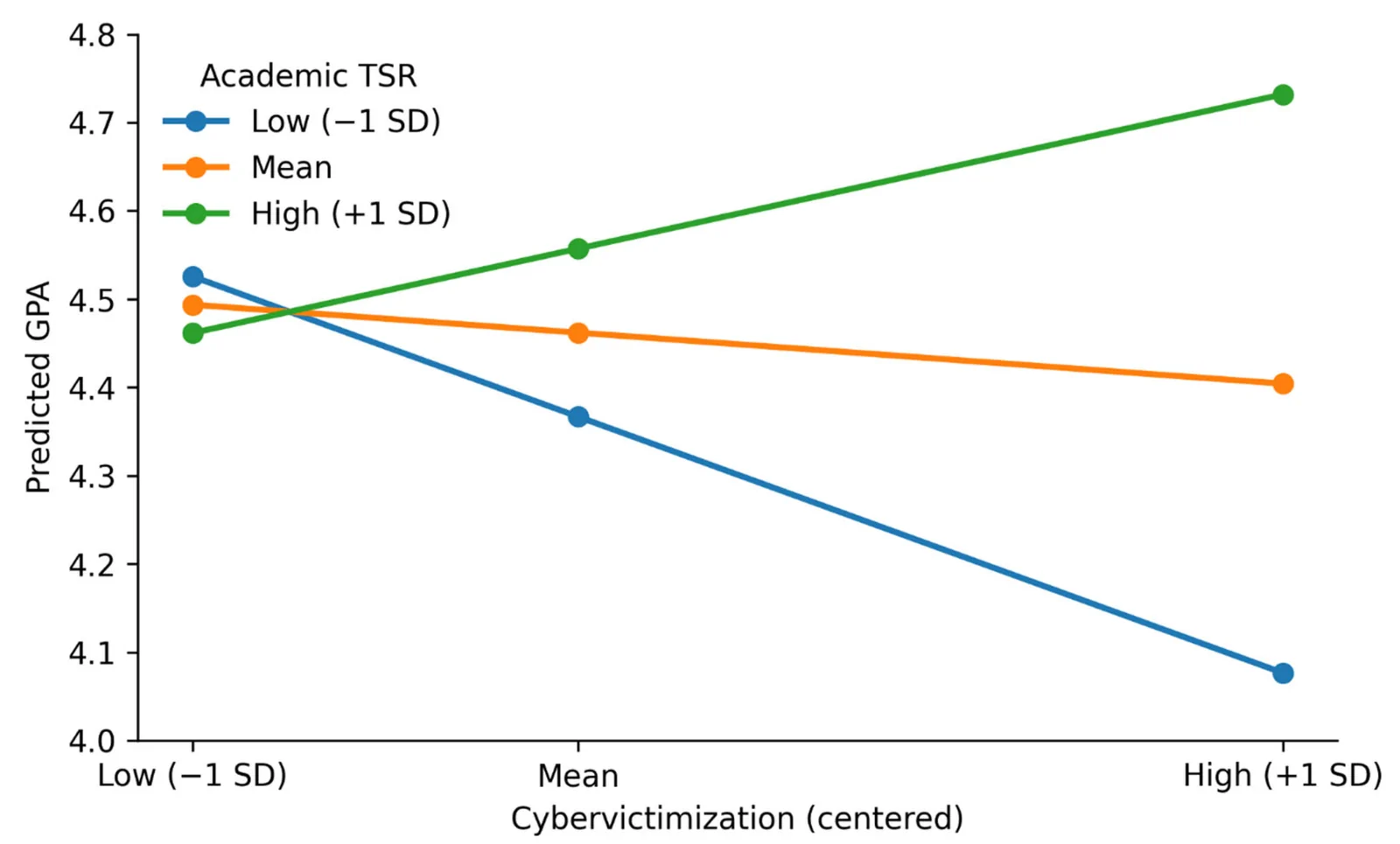
Predicted GPA as a Function of Cybervictimization and Academic TSR Level. Note. Social TSR held at its mean; predicted values derived from PROCESS Model 3 with covariates held constant.

**Table 1:** Descriptive Statistics and Pearson Correlations Among Study Variables.

	*M*	*SD*	1	2	3	4	5
1. GPA	4.47	0.93	—				
2. Cybervictimization_RCBI	12.15	3.92	−0.13 **	—			
3. Academic_Relations_STRM	50.92	12.93	0.06 *	−0.06 *	—		
4. Social_Relations_STRM	35.24	8.87	−0.01	0.03	0.71 **	—	
5. Age	14.04	0.20	0.01	−0.07 **	0.04	0.03	—

Note. GPA was coded from 1 (Not accepted/Fail) to 5 (Excellent), with higher scores indicating better academic performance. Academic TSR and Social TSR are summed scores across 15 items (possible range 15–75) and 10 items (possible range 10–50), respectively. Descriptives are based on the analytic sample (N = 1458). * *p* < 0.05, ** *p* < 0.01.

**Table 2:** Moderated regression predicting GPA (PROCESS Model 3).

Predictor	*b*	*SE*	*t*	*p*	95% CI [LL, UL]	Local *f*^2^
Constant	6.2832	1.6408	3.83	<0.001	[3.0646, 9.5018]	—
Cybervictimization (X)	−0.0147	0.0077	−1.91	0.057	[−0.0299, 0.0004]	—
Academic relations (W)	0.0074	0.0026	2.80	0.005	[0.0022, 0.0125]	—
X × W	0.0046	0.0007	6.85	<0.001	[0.0033, 0.0059]	0.033
Social relations (Z)	−0.0034	0.0039	−0.88	0.380	[−0.0110, 0.0042]	—
X × Z	−0.0008	0.0011	−0.79	0.433	[−0.0030, 0.0013]	<0.001
W × Z	0.0004	0.0002	1.86	0.064	[0.0000, 0.0007]	0.002
X × W × Z	0.0001	0.0001	1.62	0.106	[0.0000, 0.0002]	0.002
Sex (0 = male, 1 = female)	−0.0216	0.0467	−0.46	0.644	[−0.1133, 0.0701]	—
Age	−0.0503	0.1151	−0.44	0.662	[−0.2760, 0.1754]	—
Father education	0.0795	0.0489	1.63	0.104	[−0.0164, 0.1755]	—
Mother education	−0.2604	0.0683	−3.81	<0.001	[−0.3944, −0.1265]	—
Household income	−0.1792	0.0394	−4.55	<0.001	[−0.2564, −0.1019]	—

Note. Model *R*^2^ = 0.093; adjusted *R*^2^ = 0.086; *F*(12, 1445) = 12.39, *p* < 0.001. Highest-order interaction test: Δ*R*^2^ = 0.0016, *F*(1, 1445) = 2.61, *p* = 0.106. Continuous focal predictors were mean-centered prior to forming product terms. Local *f*^2^ values are reported for interaction terms; for the focal X × W interaction, partial *R*^2^ = 0.031 and local *f*^2^ = 0.033.

## Data Availability

The data presented in this study are available on request from the corresponding author due to restrictions (e.g., privacy, legal or ethical reasons).

## Abbreviations

The following abbreviations are used in this manuscript:

TSR	Teacher–Student Relationship
GPA	Grade Point Average
RCBI-II	Revised Cyber Bullying Inventory–II
